# Troponin I, CK-MB, and inotropic score in hypoxic-ischemic encephalopathy and associated infant mortality

**DOI:** 10.1186/s12887-023-04311-8

**Published:** 2023-10-16

**Authors:** Hayriye Gözde Kanmaz Kutman, Gülsüm Kadıoğlu Şimşek, Burak Ceran, Esra Beşer, Fuat Emre Canpolat

**Affiliations:** Department of Neonatology, University of Health Sciences, Ankara City Hospital MH5, 06800 Çankaya, Ankara Turkey

**Keywords:** Hypoxic-ischemic encephalopathy, Mortality, Inotropic score, Troponin I

## Abstract

**Purpose:**

Cardiovascular dysfunction is common in hypoxic-ischemic encephalopathy (HIE), which is one of the leading causes of multi-organ failure in neonates. We aimed to assess troponin I and creatine kinase myocardial band (CK-MB) levels, inotropic score (IS) in HIE patients, and their associations with HIE staging and mortality.

**Method:**

The medical records of all HIE infants admitted to our unit between 2016 and 2018 were retrospectively analyzed. Demographic characteristics of the infants, seizures, anticonvulsive therapies, maximum inotrope doses, and the derived IS (dopamine dose [µg/kg/min] + dobutamine dose [µg/kg/min] + 100 × epinephrine dose [µg/kg/min]) and CK-MB and troponin-I levels obtained in the first six hours of life were compared according to HIE staging. Comparisons between survivors and non-survivors were made.

**Results:**

The study included data from 195 patients. Twenty-five patients were classified as stage 3, 116 as stage 2, and 54 as stage 1 HIE. Median Troponin-I, CK-MB level, and IS significantly differed by HIE staging (p < 0.01). The deceased infants had significantly higher median troponin I level [0.36 (0.02-3) vs. 0.16 (0.01–1.1) ng/ml; p = 0.006], median IS [20 (5-120) vs. 5 (5–10); p < 0.001], however, CK-MB values were comparable with survivors [129 (51–300) vs. 60.7 (31–300) ng/ml; p = 0.57]. The area under the curve was 0.93 for IS and 0.81 for Troponin I to predict mortality.

**Conclusion:**

Troponin I, CK-MB, and IS could be successfully used as disease severity markers in HIE furthermore, troponin I and IS, are good predictors of mortality. These results need to be confirmed with larger prospective multi-center studies.

## Introduction

Hypoxic-ischemic encephalopathy (HIE) is the fifth leading cause of death in children under 5 years old and accounts for 23% of neonatal deaths worldwide [1]. According to Cochrane meta-analysis data, treatment with therapeutic hypothermia (TH) reduces rates of HIE mortality and significant neurodevelopmental delay at 18 months [2]. Despite TH, the mortality rate in HIE is still reported as 26%, and cardiac dysfunction is among the primary causes of death in these patients [3, 4]. In numerous randomized controlled studies, hypotension and cardiac dysfunction have been reported in a substantial proportion of HIE patients who underwent TH [5–10].

In patients with perinatal asphyxia, cardiac output is directed from less vital organs (liver, kidneys, and intestines) toward more critical organs such as the brain, heart, and adrenal gland due to hypoxia. Bradycardia develops rapidly with acute hypoxia and persists as acidosis becomes more severe; blood pressure is normal at this stage due to peripheral vasoconstriction and the redistribution of cardiac output toward vital organs. If asphyxia continues, blood pressure drops, and ischemia occurs in the vital organs. Low heart rate due to asphyxia and acidosis and the subsequent decrease in myocardial contractility due to ischemic cardiac injury reduce ventricular output and stroke volume. With reduced cardiac output, coronary perfusion decreases, and myocardial damage occurs. Troponin, a protein complex consisting of I, T, and C subunits, is located at the actin filaments of the myocardium and is essential for calcium-mediated muscle contraction. Troponin I and T are cardiac-specific subunits and markers of myocardial damage. Several studies have demonstrated that troponin-I is a good marker of myocardial dysfunction and early death in infants with HIE [9–13].

Inotropic score (IS) is a standard formula based on dopamine, dobutamine, and epinephrine dosages that was first described in the Boston Circulatory Arrest Study [14] and later used as a measure of postoperative pharmacologic cardiac support. As IS was adopted into clinical use, many studies showed that myocardial dysfunction could be a marker of disease severity, morbidities, and mortality after heart surgery; however, current knowledge is scarce about its use in HIE-associated mortality [14–18].

In addition to the previously established clinical and laboratory markers such as Troponin-I and CK-MB, we conducted this study to determine whether IS, which has not been previously used in this context, also correlates with the risk of myocardial dysfunction and mortality in HIE.

## Materials and methods

*Study Design*: This retrospective study included patients followed in our center for HIE during 2016 and 2018. All patient information was obtained from medical records. Ethical approval was obtained from the local ethics committee (No:74/2018, 13.12.2018).

### Patients

The study included infants born at a gestational age of > 34 weeks in our center or transported from another center within 6 h of birth and diagnosed with HIE. The diagnosis of HIE was made based on the presence of acute peripartum/intrapartum event with pH ≤ 7.00 or BE ≤ -16 mmol/L within the first hour after birth, Apgar score < 5 at 3–10 min or persistent need for resuscitation, and findings of moderate or severe encephalopathy on clinical assessment [19]. Infants with congenital anomalies, inborn errors of metabolism, or maternal drug addiction were excluded from the study.

### Management of HIE

In our unit, HIE diagnosis and treatment is planned based on a standard algorithm complying with the recommendations of the Turkish Neonatal Society Guideline on Neonatal Encephalopathy [19]. All infants with suspected HIE are assessed in the delivery room via cord blood gas analysis and physical examination after appropriate resuscitation. Those diagnosed with HIE are admitted to the intensive care unit. Vital signs, urine flow rate, and amplitude-integrated electroencephalography (aEEG) are monitored continuously. HIE severity is determined using the Sarnat staging criteria, and TH is initiated within the first 6 h after birth for patients with stage 2–3 HIE. In TH, the patient’s body temperature is maintained at 33–34^o^C for 72 h, followed by gradual warming at a rate of 0.5–1^o^C per hour using servo-controlled hypothermia devices (Arctic Sun ® and Tecoderm ®). All patients undergo routine laboratory tests, including blood gas analysis, troponin I, and creatine kinase MB (CK-MB) in the first 24 h of life. Both CK-MB and Troponin I are studied with a chemiluminescent assay technique, with a highly sensitive method. Cranial ultrasonography and echocardiography are performed as early as possible in the first 72 h of life.

Management of shock/hypotension: Per unit protocol, inotropic and vasoactive medications are initiated in the neonatal intensive care unit (NICU) at the attending neonatologist’s and pediatric cardiologist’s discretion. The NICU team makes decisions regarding the ongoing titration of vasoactive/inotropic medications based on each patient’s clinical condition and echocardiographic findings.

### Data Collection

The patients’ demographic characteristics, gestational age, birth weight, sex, APGAR score, delivery method, need for resuscitation, cord blood gas, HIE stage, presence of convulsions and anticonvulsive therapy received, aEEG findings, doses and duration of inotropic therapy, TH, laboratory results, length of hospital stay, and mortality data were recorded. Maximum inotrope doses were recorded and used to calculate IS (dopamine dose [µg/kg/min] + dobutamine dose [µg/kg/min] + 100 × epinephrine dose [µg/kg/min]).

### Data Analysis

SPSS version 22.0 for Windows (SPSS Inc., Chicago, Ill., USA) was used for statistical analyses. Categorical variables were reported as numbers and percentages, and continuous variables as mean ± SD or median (IQR) where appropriate. Chi-square and Fisher’s exact tests were used for categorical variables. We used the Student-t test to analyze continuous variables with normal distribution and the Mann-Whitney U test for non-normally distributed data. The One Way ANOVA test was used for group analysis for normally distributed data, and the Kruskal-Wallis H test for non-normally distributed ones. Differences were considered significant at p < 0.05 (two-tailed). Receiver operator curve analysis was performed to predict mortality.

## Results

**Fig. 1:**
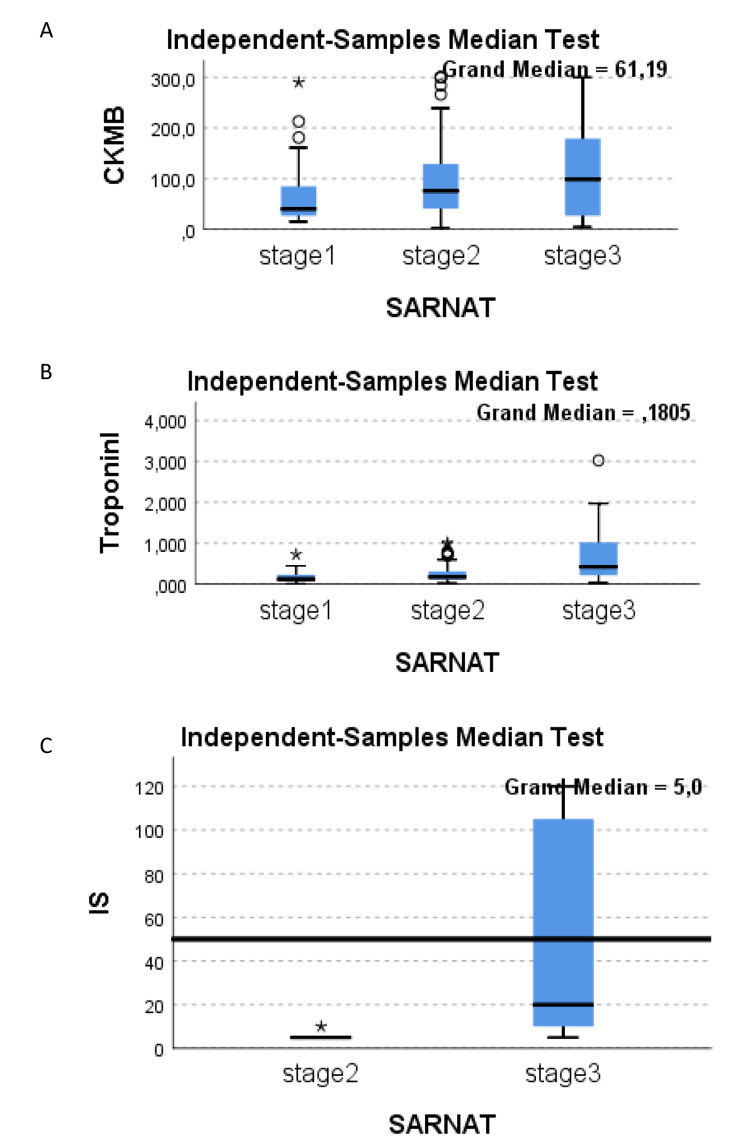
(**A**) The representation of the distribution of median CK-MB levels across the Sarnat Staging groups (**B**) The representation of the distribution of median Troponin I levels across the Sarnat Staging groups (**C**) The representation of the distribution of median IS across the Sarnat Staging groups

A total of 202 HIE patients were assessed during the study period. Seven patients did not meet the inclusion criteria and were excluded from the study. Baseline characteristics and laboratory findings of the 195 HIE patients included in the study analysis are summarized according to Sarnat&Sarnat staging in Table 1. Twenty-five (12.8%) patients were classified as stage 3, 116 (59.4%) as stage 2 and 54 (27.6%) as stage 1 HIE. Gestational age and mean birth weight were similar between the HIE staging groups (Table 1). All patients with HIE stages 2 and 3 underwent TH. Cord blood base excess, 5th minute APGAR score, cardiac compression requirement during resuscitation, need for inotropic support, seizures, and mortality rates significantly differed between HIE stages (Table 1). The number of infants that required inotrope treatment was 28 (14.3%), n = 13 (46.4%) was stage 2 HIE, and n = 15 (53.6%) was stage 3 HIE (p < 0.01). Troponin I, CK-MB levels, and maximum IS significantly increased by HIE disease severity (Table 1, Fig. 1). Median Troponin I, CKMB, and IS by every HIE stage were demonstrated in Fig. 1.

**Table 1:** Baseline characteristics and laboratory findings by hypoxic-ischaemic encephalopathy (HIE) grading

	HIE Grade I (n = 54)	HIE Grade II (n = 116)	HIE Grade III (n = 25)	p
Gestational age (weeks), mean ± SD	38.6 ± 1.7	38.5 ± 2	37.6.±2.4	0.143*
Birthweight (gram), mean ± SD	3157 ± 566	3199 ± 448	3080 ± 763	0.662*
Maternal Age, mean ± SD	25 ± 5.7	27 ± 6	29.5 ± 6.2	0.01
Gestational diabetes, n(%)	2 (0.03)	8 (0.06)	1 (0.04)	0.75
Cord blood pH, mean ± SD	7.05 ± 0.1	6.90 ± 0.1	6.79 ± 0.26	0.075*
Cord blood base deficit, mmol/L, mean ± SD	14.4 ± 3.5	18.8 ± 5.3	23.6 ± 7.2	0.001*
5th min. APGAR score Median(IQR)	8 (4–9)	7 (2–9)	3 (0–7)	0.009¥
Cardiac compression in delivery room, n (%)	1 (0.5)	4 (3.4)	18 (72)	0.0001¶
Inotrope requirement, n (%)	-	13 (11)	15 (60)	0.0001¶
Seizure, n (%)	-	36 (30)	20 (80)	0.001¶
Therapeutic hypothermia, n(%)	2 (3.8)	116 (100)	25 (100)	0.001¶
Mortality, n(%)	-	-	18 (72)	0.001¶
CK-MB, ng/ml median (IQR)	40.1 (15.3–290)	75.9 (31–300)	98.8 (51–300)	0.004*
Troponin I, ng/ml median (IQR)	0.12 (0.01–0.73)	0.17(0.01–1.01)	0.42 (0.02-3)	< 0.01*
IS median (IQR)	-	5 (5–10)	20 (5-120)	< 0.01¥

CK-MB: IS: inotropic score

*,One way ANOVA; ¥, Man-Whitney-U test; ¶ ,Chi-square

**Fig. 2:**
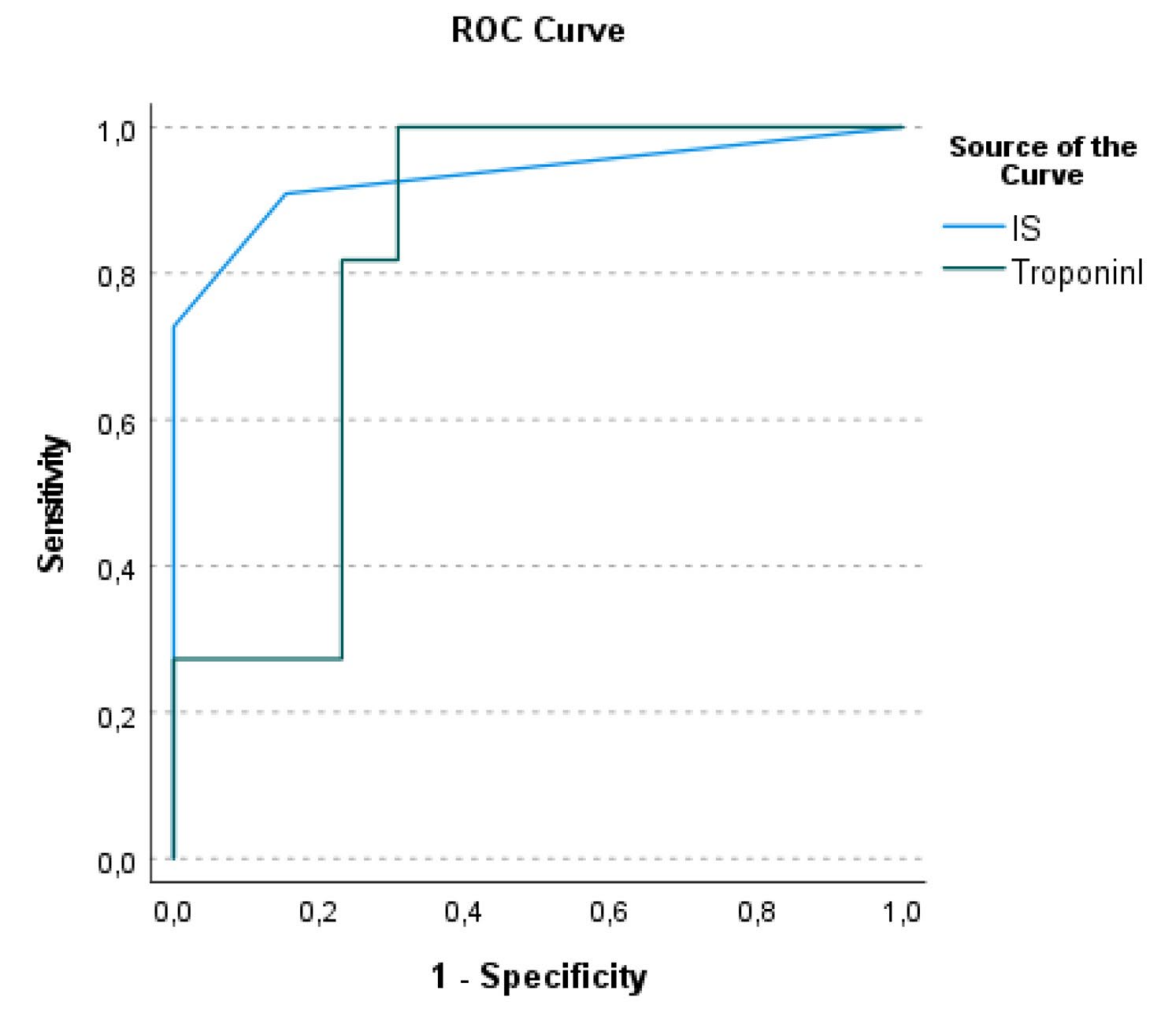
Receiver operating characteristic (ROC) curve analysis represents the effect of IS and Troponin I to predict mortality

A total of 18 infants (9.2%) died during hospitalization; all were stage 3 HIE, and the cause of death was multi-organ failure in all of them. Comparison of survivors and non-survivors showed that deceased infants had significantly higher median troponin I level [0.36 (0.02-3) vs. 0.16 (0.01–1.1) ng/ml; p = 0.006], median IS (20 [5-120] vs. 5 [4–9]; p < 0.001), while mean CK-MB values were similar between the groups [129 (51–300) vs. 60.7 (31–300) ng/ml; p = 0.57]. Cord blood base deficit, 5-minute APGAR score, cardiac compression requirement, need for inotropic support, the prevalence of convulsions, and TH frequency also significantly differed among the non-survivors (Table 2).

**Table 2:** Comparison of demographic and laboratory findings in survivors and non-survivors

	Survivors (n = 177)	Non-survivors (n = 18)	p
Gestational age (weeks), mean ± SD	38.5 ± 1.9	37.6 ± 2.5	0.08*
Birthweight (gram), mean ± SD	3187 ± 506	2973 ± 686	0.11*
Cord blod pH, mean ± SD	6.9 ± 0.5	6.8 ± 0.28	0.358*
Cord blood base deficit, mmol/L, mean ± SD	17.7 ± 5.6	21.9 ± 7.2	0.008*
5th min. APGAR score, median(IQR)	7 (2–9)	4 (0–7)	0.009^¥^
Cardiac compression in the delivery room, n (%)	12 (6.7)	11 (61)	< 0.001^¶^
Inotrope requirement, n (%)	15 (8.5)	13 (72)	< 0.001^¶^
Seizures, n (%)	43 (24)	13 (72)	< 0.001^¶^
Therapeutic hypothermia, n(%)	126 (71)	18 (100)	0.004^¶^
CK-MB, ng/ml, median(IQR)	60.7 (31–300)	129 (51–300)	0.57^¥^
Troponin I, ng/ml, median(IQR)	0.16 (0.01–1.1)	0.36 (0.02-3)	0.006^¥^
Maximum IS, median(IQR)	5 (5–10)	20 (5-120)	< 0.001^¥^

IS: inotropic score

¶ Chi-square * Students t-test, ¥ Man-Whitney-U test

Furthermore, the median CKMB [75.9 (31–300) vs. 40.1 (15–290) ng/ml; p = 0.002] and Troponin I [0.19 (0.01-3) vs. 0.12 (0.01–1.72) ng/ml; p = 0.029], was significantly higher in infants who underwent therapeutic hypothermia compared to infants who did not get therapeutic hypothermia.

Receiver operating characteristic (ROC) curve analysis was performed for markers that may predict mortality in neonates with HIE. Both IS (standard error [SE]: 0.05; 95% CI: 0.81–1.01, p < 0.01) and Troponin I was found to be sensitive predictors of mortality with an area under the curve (AUC) of 0.968 and 0.81 (standard error [SE]: 0.09; 95% CI: 0.63–0.99, p = 0.008) (Fig. 2). The optimal cut-off value for troponin I in predicting mortality was 0.249 ng/ml (90% sensitivity, 70% specificity).

## Discussion

This retrospective analysis demonstrated that troponin I and inotropic score significantly predict mortality in infants with HIE. Furthermore, CKMB and Troponin are also associated with the severity of HIE. Several studies have shown that the severity of HIE has been associated with many biomarkers (6–10). Levels of CK-MB began to rise within the first few hours of life and are significantly higher in moderate and severe grades of neonatal hypoxic ischemia compared with mild grades and normal controls within the first 2–4 h. However, CK-MB does not appear to discriminate well between those infants with and without cardiovascular compromise ([6, 20–22]). Similarly, we found that CKMB levels were significantly lower in mild HIE patients; however, there was no statistical difference between infants who survived HIE and those who did not.

Troponin I is a good indicator of myocyte damage; serum levels in healthy individuals are undetectable. Previous reports confirmed a definite correlation between the HIE stage and troponin I level [11–13]. In our study, troponin I level increased with the HIE stage, and a cut-off value of 0.249 ng/mL was determined for mortality. Moreover, Troponin I levels were significantly higher in patients who underwent therapeutic hypothermia since the infants with Stage 2 and 3 underwent therapeutic hypothermia. The design of the current study cannot explain whether the higher troponin-I levels were caused by therapeutic hypothermia or the HIE stage. Türker et al. [13] identified cord cardiac troponin I as the most sensitive factor in predicting early death, with a cut-off value of 4.6 ng/mL; however, unlike our results, they found that CK-MB was also significant in predicting mortality. In another study, the troponin I cut-off value for predicting mortality was 8.1 mg/ml [16]. The different cut-off values reported in the literature may be due to differences in sampling time. Kanık et al. reported that troponin-I obtained at postnatal 72 h was a good predictor of mortality, while CK-MB did not have predictive value [5]. Our results confirmed previous studies however, to our knowledge, it has the largest sample size, which includes 141 moderate to severe HIE infants.

Cardiac dysfunction results in reduced perfusion, tachycardia, hypotension, and the need for inotropic support. Troponin I level, shown to predict myocardial injury and cardiac dysfunction in HIE, was strongly correlated with the duration of inotropic support in these patients [12]. Limited knowledge of the relationship between inotropic support duration and HIE disease severity, prognosis, and mortality exists. There are no data regarding the measurement of inotropic support using a standard formula based on dose and drug potency and its association with prognosis. In pediatric heart surgery patients, low cardiac output syndrome and maximum IS were strongly correlated with mortality, mechanical ventilation time, and length of hospital stay [14–17]. Our study demonstrated that maximum IS increased by the HIE stage and changed significantly with troponin I in surviving and non-surviving HIE infants. In several previous studies, troponin-I has the highest predictive mortality value in HIE patients [9, 11–13]. This study is the first to study IS in infants with HIE, demonstrating its superiority to troponin I in predicting mortality (AUC 0.96). Recently Malai et al. reported that a need for two or more inotropic drugs (aOR 45.7, 95% CI 1.5–1040, *p* = 0.029) was a significant factor for mortality in a relatively large cohort of HIE neonates, however unlike us, they did not study the inotropic scores [17].

The main limitation of the study was its retrospective design. Echocardiographic examinations and troponin-T levels were not included, which could better indicate cardiac injury in neonates with HIE. Vasoactive inotrope scores (VIS) could also be studied in this study however, unfortunately, vasopressin, which is in the VIS equation, is unavailable in Turkey. These results must be supported with a larger group of infants with a more significant number of alive infants with Grade 3 HIE who required inotropic treatment. Furthermore, we thought that a relatively low number of dead infants may have altered the results.

It may be challenging to predict mortality within the first 24 h in neonates with HIE; based on these results and previous others in the literature, assessment of early troponin I level and monitoring IS will help manage infants with a high risk of mortality appropriately.

## Conclusion

Our study is the first to use IS to predict mortality in infants with HIE. Based on our results, mortality due to circulatory failure is significantly higher. Further prospective and larger studies are required to support our findings and to define the association of this scoring system with other morbidities and long-term outcomes.

## Data Availability

The datasets used and/or analyzed during the current study are available from the corresponding author on reasonable request.
